# Cholera

**Published:** 1892-09

**Authors:** R. M. Swearingen

**Affiliations:** State Health Officer; Austin, Texas


### CHOLERA.

That cholera will be imported into the United States is a foregone conclusion; that it will become epidemic is extremely doubtful.

Since its last appearance amongst us much has been learned of its cause, and therefore it is pretty well understood how to manage it. It is known to be propagated by means of a pathogenic germ, whether Koch's comma bacillus or not does not much matter; and that this poison is contained almost entirely in the alimentary canal of those suffering an attack. The danger, therefore, is in the *dejecta* of those sick with the disease. Knowing where and what the danger is it would seem any easy matter to avoid it.

While the disease cannot originate in filth or in crowds, these serve as fuel to light up a conflagration when the spark—the cholera germ—is introduced. Hence the necessity of cleaning up.

The greatest source of danger is the water. Cholera *dejecta*, the vomit and stools, if thrown on the ground or in privy vaults, readily contaminate the water in streams or wells, or in cisterns if the cement be cracked, even at a distance; hence the recommendation to boil before using, all water for domestic purposes. Hence the popular but fallacious idea that a looseness of the bowels is a forerunner of cholera, or that diarrhœa turns to cholera; and the caution to abstain from certain articles of food. There is no danger of food if the water in which it is cooked is not contaminated with the cholera germ. All filthy places should be cleaned up. Then, if a cholera case breaks out amongst us, isolate it, and receiving every particle of the *dejecta* in a vessel which will close up, take it out and burn it; or, add to it a strong solution of bichloride of mercury.

The *standard solution* for disinfecting feces is, corrosive sublimate 4 oz., sulphate of copper 1 pound, to a gallon of water; of this eight ounces should be used for each stool. Should vomit or other dejecta get on clothing, sheets, or any other fabric, however costly, it should be burned. If on the carpet run a very hot iron over it, after disinfecting the spot with the bichloride.

With these precautions observed, no one need fear cholera, even should it be imported into Texas.

MINDING THE GAPS.—State Health Officer Swearingen is fully alive to the importance of taking necessary steps to protect Texas from cholera. At every seaport a strict quarantine is in force, and the inspection service on the Mexican border has been doubled. Inspectors have been appointed for all railroads entering Texas, and should cholera break out anywhere in the United States and it become necessary to quarantine, these inspectors will be put on all incoming trains, and no one who has been in an infected town, or any place reported to be infected, will be allowed to enter the State. All is now in readiness to establish camps of observation and put on inspectors at a moment's notice.

A TIMELY WARNING.—The following circular letter has been been sent to every local health officer in Texas and published in most of the newspapers:

"AUSTIN, TEXAS, September 1, 1892.

"DEAR DOCTOR—The rapid spread of cholera from Russia to Germany, thence to France and England, admonishes those familiar with its history that the disease has begun its tour around the world.

"During the last quarter century while cholera has been dormant in its natural home, with an occasional outbreak in Eastern cities, maritime quarantine and sanitary science have been advancing; and we are better prepared than ever before to measure strength with this formidable enemy.

"To the county health officers and city physicians of this State, who constitute the army of defense, the State health officer respectfully submits the following suggestions:

"The State quarantine authorities have already instituted vigorous measures to prevent the introduction of the disease through any of our sea-ports into Texas; but the extensive border lines to be guarded, should it secure a foot-hold in any of the other States, or in Mexico, renders the chances of exclusion by quarantine extremely doubtful.

"This threatening danger calls for the exercise of unceasing vigilance on the part of the health authorities, and an intelligent and cordial co-operation by the people. Should it elude the vigilance of our sentinels on the outposts, sanitary science must be invoked to stay its ravages.

"Cholera is essentially a filth disease; and while it may not originate *de novo* in filth, it is certain that unsanitary conditions favor its rapid propagation; hence the necessity for a rigid observance of the laws of hygiene, public and private, general and personal.

"It is known that the dejecta of cholera patients constitute the principal source of infection, and that it is through this medium that the atmosphere and the water become contaminated. Pure drinking water is of paramount importance. Springs, wells, creeks and rivers are easily polluted, either by seepage from vaults, or by the washings of the surface where these dejecta are too often thrown through ignorance of their danger. Whenever pollution of any water supply is suspected the water for domestic purposes should be boiled before being used.

"Cleanliness, absolute and continued, should be maintained in and about every home, upon every farm, in every village, town and city in the State. All privy vaults should be filled up with dirt, and portable boxes or tubs should be substituted for them; these can be easily emptied and made innocuous by the free use of lime, zinc or bichloride of mercury. It is an established fact that privy pits are capable of contaminating with deadly effect wells, and running streams, even miles away from them. Health officers of cities and incorporated towns are strongly urged to impress, with emphasis, this important fact upon the authorities, and to insist that their respective city councils at once, by suitable ordinance require all underground sinks and pits to be immediately filled up and abandoned; a severe penalty being imposed in every case for non-compliance with said ordinance.

"Should city councils refuse or fail to enact such ordinance, appeal from them to the people. If every citizen could be made to appreciate the importance of setting his own house in order, and be induced to do it, epidemics would be of short duration, and the death rate greatly lessened.

"I trust and believe that every health officer will feel the responsibility resting upon him, and come up to the full measure of his duty.

"I have the honor to be your obedient servant,

"R. M. SWEARINGEN, State Health Officer."

